# The positive impact of red palm oil in school meals on vitamin A status: study in Burkina Faso

**DOI:** 10.1186/1475-2891-5-17

**Published:** 2006-07-17

**Authors:** Augustin N Zeba, Yves Martin Prével, Issa T Somé, Hélène F Delisle

**Affiliations:** 1 Department of Nutrition, Faculty of Medicine, Université de Montréal, C.P. 6128 succ. Centre-ville, Montréal Qc, H3C 3J7, Canada; 2 (IRSS) Institut de Recherche en Sciences de la Santé/DRO, 01 BP 545 Bobo Dioulasso 01, Burkina Faso; 3 IRD (Institut de recherche pour le développement), Unité de Recherche 106 «Nutrition, Alimentation, Sociétés», 01 BP 182 Ouagadougou 01, Burkina Faso; 4 Laboratoire de chimie analytique et de toxicologie, UFR Sciences de la santé, Université de Ouagadougou, 03 BP 7021, Ouagadougou 03, Burkina Faso

## Abstract

**Background:**

Vitamin A (VA) deficiency is widespread in sub-Saharan Africa and school-age children are a vulnerable group. In Burkina Faso, the production and consumption of red palm oil (RPO) is being promoted as a food supplement for VA. The objective of the study was to assess the impact on serum retinol of adding RPO to school lunch in two test zones of Burkina Faso.

**Methods:**

Over one school year, 15 ml RPO was added to individual meals 3 times a week in selected primary schools in two sites. Serum retinol was measured with HPLC at baseline and exactly 12 months later to take account of seasonality. A simple pre-post test design was used in the Kaya area (north-central Burkina), where 239 pupils from 15 intervention schools were randomly selected for the evaluation. In Bogandé (eastern Burkina), 24 schools were randomised for the controlled intervention trial: 8 negative controls (G1) with only the regular school lunch; 8 positive controls (G2) where the pupils received a single VA capsule (60 mg) at the end of the school year; and 8 schools with RPO through the school year (G3). A random sample of 128 pupils in each school group took part in the evaluation.

**Results:**

In Kaya, serum retinol went from 0.77 ± 0.37 μmol/L at baseline to 1.07 ± 0.40 μmol/L one year later (p < 0.001). The rate of low serum retinol (<0.7 μmol/L) declined from 47.2% to 13.1%. In Bogandé, serum retinol increased significantly (p < 0.001) only in the capsule and RPO groups, going from 0.77 ± 0.28 to 0.98 ± 0.33 μmol/L in the former, and from 0.82 ± 0.3 to 0.98 ± 0.33 μmol/L in the latter. The rate of low serum retinol went from 46.1 to 17.1% in the VA capsule group and from 40.4% to 14.9% in the RPO group. VA-deficient children benefited the most from the capsule or RPO. Female sex, age and height-for-age were positively associated with the response to VA capsules or RPO.

**Conclusion:**

RPO given regularly in small amounts appears highly effective in the reduction of VA deficiency. RPO deserves more attention as a food supplement for VA and as a potential source of rural income in Sahelian countries.

## Background

Vitamin A (VA) deficiency affects approximately 40% of the world population, particularly pregnant or lactating women and under-five children [1]. An estimated 100–140 million children are still suffering from subclinical VA deficiency, although clinical signs of the deficiency are on the decline [1]. Even subclinical VA deficiency is associated with 23% excess mortality of under-five children [2] and with maternal mortality [3]. Sahelian countries, including Burkina Faso, are the most affected by VA deficiency in sub-Saharan Africa. In a small community-based study conducted in 1999 in the north-central part of Burkina Faso, 84.5% of under-five children and 61.8% of their mothers were VA-deficient according to serum retinol concentrations [4].

Large scale periodic supplementation of under-five children with high-dosage VA capsules is still the preferred VA strategy in most developing countries (DCs) [5]. Several studies have shown the efficacy of community- or hospital-based VA supplementation [2,6,7]. However, it should only be a short-term approach to control the deficiency as it is not sustainable. Supplementation tends to entertain dependency and to convey the idea that VA deficiency is a medical problem, not a food and nutrition problem, which it is [8]. VA fortification is used as a preventive measure even in high-income countries. A severe limitation in many low-income countries is that it is hard to identify appropriate food vehicles for fortification [9,10]. Dietary diversification is a sustainable and long-term approach to the control of VA deficiency. Dietary diversification refers to several types of food system-based interventions designed to increase the supply, distribution and consumption of VA-containing foodstuffs [11]. While animal sources of VA in the form of retinol are highly bio-available, their access is often constrained by poverty. There is a wide variety of plant sources of provitamin A carotenoids, but their availability is often seasonal and their bio-efficacy may be quite low as in the case of green leaves [12,13].

Red palm oil (RPO) is the highest plant source of provitamin A carotenoids, and it is highly bio-available because of the fat milieu and the absence of a plant matrix [14-16]. RPO is not only a source of VA; it provides fat, which is often in short supply and affects the bio-efficacy of dietary provitamin A carotenoids. RPO is also a source of several antioxidants including vitamin E and non-VA carotenoids which are involved in the prevention of cancer and other chronic diseases [17-19]. RPO has been shown, contrary to common belief, to have a protective role in cardiovascular disease through increasing HDL-cholesterol [20,21]. It is a staple fat in several countries of West, Central, and Southern Africa. However, the levels of consumption by nutritionally vulnerable groups and the extent of oil blanching, which destroys the VA activity, are by and large unknown. Many trials and a few programmes showed the efficacy and effectiveness of RPO in children and in women [22-29]. In Burkina Faso, it was shown in a pilot-study that it was possible through social marketing to bring people who were unaccustomed to RPO to purchase and consume it to protect women and children from VA deficiency [4]. After two years of promotional activities, it was found that around 45% of women and children in the test area had consumed RPO in the week prior to the survey [30]. This led to a substantial decline in the rate of low serum retinol in the study area, from 61.8% to 28.2% in mothers, and from 84.5 to 66.9% in children [4]. Based on market studies, it was also concluded that in the western part of Burkina Faso, RPO could be produced in much larger amounts by women who traditionally extract the oil, provided there is a demand for the product.

Following the pilot study, a larger project was implemented and one of the components was the RPO-fortification of school meals in selected areas. The school intervention was designed as a means of improving VA status of pupils and as a channel for RPO introduction in communities. The school lunch program is a promising entry point for nutritional improvement and for reaching communities, although not all children are enrolled in school – the rate of primary school enrolment in Burkina Faso is 27% [31]. The RPO retail system was developed so that the sites with the school intervention could have access to RPO through commercial channels. The purpose of this paper is to report on the impact of the school intervention on school children's retinol status.

## Methods

### Evaluation protocol

Impact evaluation, based on serum retinol concentration changes, was conducted in two areas where school lunches were fortified with RPO over one school year: Kaya Department (Sanmatenga province), in the north-central part of Burkina Faso, and the Bogandé District (Gnagna Province) in the eastern part. The intervention consisted of adding 15 ml RPO to individual meals 3 times a week in selected primary schools with a school lunch program in operation. This amount of RPO provides approximately 1500 μg retinol activity equivalents (RAE), based on analyses of local RPO. Parents and teachers were well informed of the purpose of the project and school lunch cooks were trained prior to implementation. Serum retinol was measured with HPLC at baseline and exactly 12 months later to take account of seasonality of VA intake and infectious diseases, which may affect VA status.

**Figure 1:**
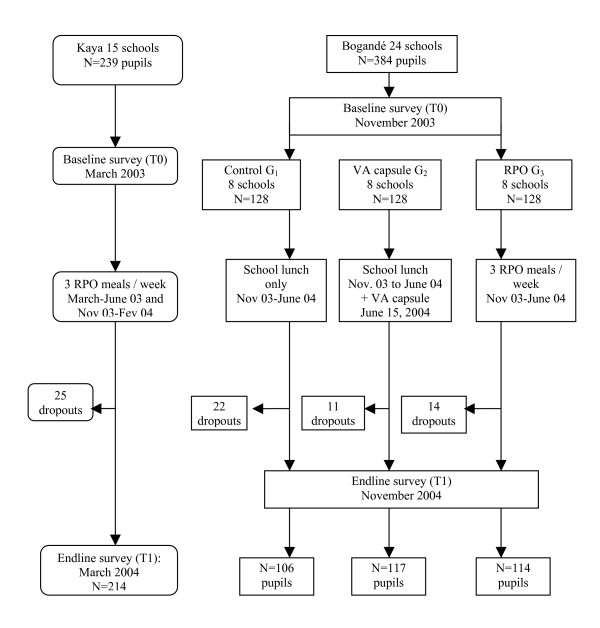
Study pupils in Kaya and Bogandé.

In Kaya, a simple pre-post test design was used. Of the 25 schools located in this district and participating in the RPO intervention, only 15 were selected for the evaluation. The remaining ones were excluded because they are located in villages exposed to the RPO intervention during the pilot study [4]. These schools had an operational school lunch program, and were distant at the most by 60 km from Kaya. Between March 2003 and March 2004, school lunches were fortified with RPO for an average of 9 weeks (mean of 28.4 ± 10.6 fortified meals); not all schools started RPO fortification at the same time. In each school, an initial number of 15 pupils was defined and a 10% allowance was included in order to take account of pupils who were to be lost to the repeat survey. The number of selected children was weighted in the schools and classes with a higher number of children, and the children in the last primary school year were excluded. A total of 239 pupils aged from 84 to 144 months were randomly selected using school lists of pupils. Blood samples for serum retinol determination were collected before the RPO fortification started in March 2003, and again in the same children in March 2004. In Bogandé (eastern Burkina), the 24 schools of the district having a school lunch program were included and were randomised for the controlled intervention trial: 8 negative controls (G_1_) with only the regular school lunch; 8 positive controls (G_2_) where the pupils received a single VA capsule (60 mg) at the end of the 2003–4 school year; and the last 8 schools with RPO from November 2003 until June 2004 (G_3_). In G_3 _schools, 50.9 ± 16.7 RPO fortified meals were served (average of 17 weeks). The same weighting and exclusion criteria procedure in the determination of selected children was used as in Kaya. It should be mentioned that in all school lunch programmes in the country, VA fortified vegetable oil is used, but the fortification level is quite low: 9 mg retinol/kg oil. Serum retinol was measured in a random sample of 128 pupils in each school group in November 2003, and again in the same pupils one year later (See Figure 1). Sample size determination in both sites was based on an expected baseline rate of 45% of low serum retinol (<0.7 μmol/L) and a post-intervention rate of 20%, with an alpha-type error of 0.05 and a statistical power of 0.80, based on prior studies among school-children in Niger, a neighbouring Sahelian country [32].

The study was approved by the Ethics Committee of the Medical School of Université de Montréal and by the Ministry of Research in Burkina Faso. An informed consent form was signed by the father before enrolling the child.

### Study variables

For each pupil, sex, age and the occurrence of illness in the previous fortnight were recorded. Weight and height were measured, and blood samples were collected, at baseline and in the repeat survey. After centrifugation on-site, serum samples were frozen and kept at -32°C until analysed in duplicate for retinol by HPLC in the Toxicology and Analytical Chemistry laboratory of the Health Science Research and Training Unit, University of Ouagadougou (Burkina Faso). This laboratory belongs to a worldwide network of labs measuring retinol and carotenoids. Low and very low serum retinol cut-offs (<0.7 μmol/L and <0.35 μmol/l, respectively) are as recommended by WHO [33].

### Data processing and analysis

Data were entered twice and analysed using SPSS 11.0., and Epi Info™, version 3.3.2 was used to compute Z-scores for anthropometric indices: height-for-age and BMI-for-age. BMI for age was used instead of weight-for-height because most pupils were above the height and age limit for weight-for-age in the repeat survey. Student t tests and khi^2 ^tests were used to compare continuous and categorical variables, respectively. Only data from children who took part in both surveys were included in the analyses. Analyses of variance with repeat measures were performed, with "treatment" as a co-factor in Bogandé school data.

## Results

### Characteristics of study subjects

As shown in Figure 1, 214 of the 239 pupils of Kaya taking part in baseline study were also included in the 2^nd ^survey one year later. In Bogandé, 337 of the initial sample of 384 pupils took part in the 2^nd ^survey. No death occurred in the study children. In 90% of the cases, the children were not available for the 2^nd ^survey because they had to help their parents in the fields. There were a few cases of pupils being transferred to another school. Mean age at baseline was 101–102 months, except G_1 _in Bogandé, where the children were younger (94 ± 20 months, see Tables 1 and 3). The percentage of girls was significantly lower than that of boys in Kaya schools only (43% against 57%, p = 0.003).

**Table 1:** Characteristics of Kaya pupils and serum retinol changes

	**Baseline (T0)**	**Endline (T1)**	**p value**
**(n = 214)**			
% girls	43.0	-	-
Age (months)	102 ± 19	-	-
Height-for-age Z-score	-0.045 ± 1.40	-0.15 ± 1.36	0.013
BMI-for-age Z-score	-1.0 ± 0.89	-0.99 ± 1.0	0.80
Serum retinol (μmol/L)			
Total	0.77 ± 0.37	1.07 ± 0.4	<0.001
Girls	0.79 ± 0.37	1.16 ± 0.45**	<0.001
Boys	0.78 ± 0.37	0.99 ± 0.34**	<0.001
Serum retinol <0.70 μmol/L (%)			
Total	47.2 (40.5–53.9)	13.1 (8,6–17.6)	<0.001
Girls	46.7 (36.7–56.7)	8.7 (3–14.4) ‡	<0.001
Boys	47.5 (38.7–56.3)	16.4 (9.9–22.9) ‡	<0.001
Serum retinol <0.35 μmol/L (%)	15.0(8.0–22.0) ‡‡	0	-
Mean serum retinol change in <0.7 μmol/L subjects at baseline n = 101 (%)	-	48.7^† ^± 23.5	-
Mean serum retinol change in ≥0.70 μmol/L subjects at baseline n = 113 (%)	-	-10.0^† ^± 43.4	-

Mean ± SD or % (CI 95%)

** At T1, mean serum retinol significantly higher in girls than in boys (p = 0. 02)

‡ At T1, rate of low serum retinol tends to be lower in girls (p = 0.098)

‡‡ The rate of very low serum retinol not different in boys and girls (p = 0.20)

† Change = [100*(Serum retinol T1 – serum retinol T0)/Serum retinol T0], significantly higher (p < 0.001) in deficient subjects at baseline

### Serum retinol changes and associated factors in Kaya

In Kaya pupils (Table 1), serum retinol increased significantly from 0.77 ± 0.37 μmol/L at baseline to 1.07 ± 0.40 μmol/L one year later (p < 0.001). The rate of low serum retinol declined accordingly, going from 47.2% at baseline to 13.1% in the 2nd survey (p < 0.001). This represents a reduction of 72%. Furthermore, 15% had very low serum retinol concentrations at baseline, but none one year later. At baseline, there was no difference between boys and girls; after the intervention, mean serum retinol was significantly higher and the rates of low serum retinol significantly lower in girls than in boys. Children with low serum retinol at baseline improved the most, with a change of 48.7 ± 23.5%, compared to -10.0 ± 43.4% in those with normal serum retinol at baseline. Table 1 also shows that the height status of the children declined significantly (p = 0.013) between the two surveys, while little change was observed for the BMI Z-score.

The multivariate analysis of Kaya data (Table 2) showed that age in months (p = 0.001), female sex (p = 0.045) and initial height-for-age Z-score (p = 0.012) were independent within-subject predictors of endline serum retinol. Between subjects, only female sex (p = 0,024) was significantly associated with a higher level of serum retinol. Baseline serum retinol was also a highly significant predictor of change (p < 0.001).

**Table 2:** Factors associated with serum retinol changes in Kaya pupils (ANCOVA with repeated measures)

**Within subject effects**				
**Source**	**Sum of squares**	**df**	**F**	**P value**

Time factor	0.89	1	6.30	0.013
Time factor* age	1.60	1	11.58	0.001
Time factor* height-for-age Z-score	0.88	1	6.39	0.012
Time factor* sex	0.56	1	4.08	0.045
Error	28.74	210	-	-

Among the Kaya pupils, 18% had received a VA capsule of 60 mg during the "National Micronutrient Days" six months earlier. Those pupils were younger that the ones who had not received the VA supplement. This is understandable since the Micronutrient days target under-five children primarily, although a few older children may slip among recipients. However, those pupils having taken a VA capsule were not different from the others for retinol or anthropometric status at baseline.

### Serum retinol changes in intervention and control groups in Bogandé

**Figure 2:**
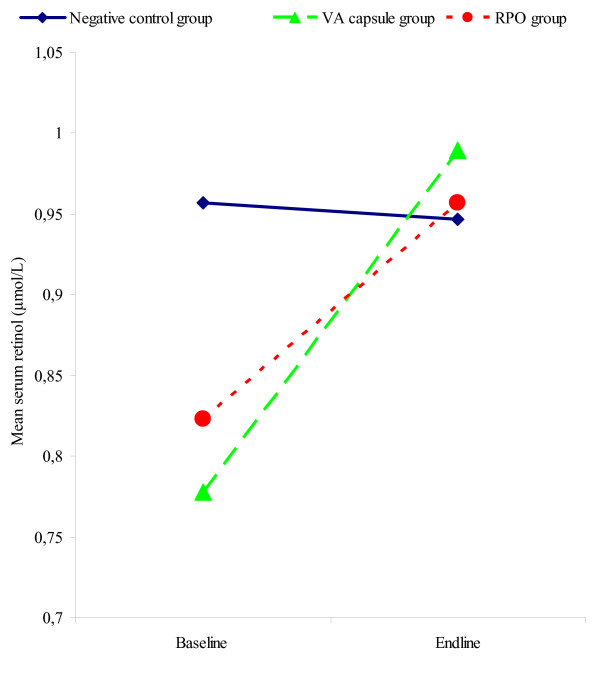
**Mean serum retinol changes according to treatment group, Bogandé**. The continuous and blue line represents the negative control group of 8 schools, where the pupils received only the regular school lunch during the 2003–04 school year. The dashed green line represents the positive control group of 8 schools, where the pupils received a single VA capsule (60 mg) at the end of the 2003–4 school year. The dashed red line represents the last 8 schools where fortified RPO meals were served to pupils from November 2003 until June 2004.

Serum retinol values in Bogandé school children are provided in Table 3. It is seen that serum retinol did not change in the negative control group (G1). This is in contrast with the significant increase in both the VA capsule (G2) and the RPO (G3) groups. Mean serum retinol went from 0.77 ± 0.28 μmol/L to 0.98 ± 0.33 μmol/L in the capsule group, and from 0.82 ± 0.30 μmol/L to 0.98 ± 0.33 μmol/L in the RPO group. The serum retinol changes in the three groups are illustrated in Figure 2.

Much like in Kaya, a higher serum retinol increase was observed in the subjects who were deficient at baseline. Among deficient subjects at baseline, the change went from +37.7 ± 47.1% in negative controls to +63.1 ± 65.7% in the RPO group and +87.5 ± 111.2% in the VA capsule group.

It is also seen in Table 3 that mean baseline serum retinol was significantly higher in the negative control group compared to the other groups in spite of the random assignment of schools to treatment groups. Negative control children were also significantly younger and had a better height status than the other groups. However, mean serum retinol differences between groups were not significant in the 2nd survey round. The anthropometric status declined in all groups in the 2nd survey, particularly BMI Z-scores. It should be noted that Bogandé school children did not receive VA capsules at any time prior to the study.

The results of the analysis of covariance with repeated measures for Bogandé children are given in Table 4. It shows that serum retinol evolved differently according to school group (p < 0.001), which was to be expected, but independent of sex. Age (p < 0.001) and height status (p = 0.009) enhanced the upward changes, as was observed in Kaya. In other words, the intervention tended to have more of an impact in older and taller children.

**Table 3:** Characteristics of Bogandé pupils and serum retinol changes

	**G_1 _(Controls)**			**G_2 _(VA capsule)**			**G_3 _(Red palm oil)**		
	
	**Baseline (T0)**	**Endline (T1)**	**p value**	**Baseline (T0)**	**Endline (T1)**	**p value**	**Baseline (T0)**	**Endline (T1)**	**p value**
**Total**	**(n = 106)**			**(n = 117)**			**(n = 114)**		

% girls	43 (34–52)	-	-	49.0 (40–58)	-	-	49.0 (40–58)	-	-
Age (months)	94 ± 20	-	-	101 ± 20	-	-	102 ± 22	-	-
Height-for-age Z-score	††0.55 ± 1.90	††0.43 ± 1.6	0.61	†† -0.16 ± 1.50	†† -0.33 ± 1.20	0.33	††-0.38 ± 1.56	†† -0.33 ± 1.20	0.78
BMI-for-age Z-score	-0.91 ± 0.94	-1.25 ± 1.27	0.022	-0.93 ± 0.96	-1.34 ± 1.00	0.002	-1.13 ± 0.88	-1.40 ± 0.99	0.03

Mean serum retinol (μmol/L)									

Total	**0.96 ± 0.36	0.94 ± 0.30	0.66	**0.77 ± 0.28	0.98 ± 0.33	<0.001	**0.82 ± 0.30	0.98 ± 0.33	<0.001
Girls	0.96 ± 0.35	0.99 ± 0.30	0.66	0.77 ± 0.29	1.00 ± 0.34	<0.001	0.83 ± 0.30	0.96 ± 0.26	0.010
Boys	0.95 ± 0.38	0.91 ± 0.30	0.50	0.78 ± 0.27	0.97 ± 0.32	<0.001	0.81 ± 0.29	0.96 ± 0.26	0.020

Serum retinol <0.70 μmol/L (%)									

Total	‡23.6 (15.6–31.6)	14.2 (8.2–20.2)	0.08	‡46.1 (36.7–56.7)	17.1 (10.1–27.1)	<0.001	‡40.4 (31.4–49.4)	14.9 (8.9–20.9)	<0.001
Girls	‡‡15.5 (5.5–25.5)	13.3 (4.3–22.3)	0.76	‡‡45,6 (33.6–57.6)	14.0 (5.0–23.0)	<0.001	‡‡36.4 (24.4–48.4)	9.1 (2.1–16.1)	<0.001
Boys	‡‡25.5 (15.5–35.5)	14.8 (6.8–22.8)	0.24	‡‡46.7 (34.7–58.7)	20.0 (10.0–30.0)	0.002	‡‡44.1 (32.1–56.1)	20.3 (10.3–30.3)	0.006

% Serum retinol <0.35 μmol/L	0.0	2.0	-	0	1.0	-	0	0	-

Mean serum retinol change in <0.7 μmol/L subjects at baseline (%)									

	-	†38.0 ± 47.1 **N = 25**	-	-	†87.5 ± 111.2 **N = 54**	-	-	†63.1 ± 65.7 **N = 46**	-

Mean serum retinol change in ≥0.70 μmol/L subjects at baseline (%)									

	-	-2.0 ± 29.0 N = 81	-		9.6 ± 33.9 N = 63	-	-	6.9 ± 39.2 N = 68	-

Mean ± SD or % (CI 95%)

** At T0, mean serum retinol significantly higher in group G_1 _than in G_2 _and G_3 _(p < 0.001)

‡ At T0, rate of low serum retinol significantly lower in group G_1 _than in G_2 _and G_3 _(p = 0.012)

‡‡ The rate of very low serum retinol not different in boys and girls in the three groups G_1_, G_2 _and G_3 _(p = 0.83, 0.90 and 0.40, respectively)

†Change [100*(Serum retinol T1 - serum retinol T0)/Serum retinol T0]: Significantly higher in initially deficient subjects of groups G_2 _and G_3 _than in G_1 _(p < 0.001)

††At T0 and T1, the pupils from G_1 _were significantly taller than those in G_2 _and G_3 _(p < 0.001)

**Table 4:** Factors associated with serum retinol changes in Bogandé pupils (ANCOVA with repeat measures)

**Within subject effects**				
**Source**	**Sum of squares**	**df**	**F**	**p**

Time factor (T0 – T1)	0.78	1	12.60	<0.001
Time factor* age	1.45		23.50	<0.001
Time factor * height-for-age Z-score	0.42	1	6.87	0.009
Time factor * sex	0.10	1	1.65	0.20
Time factor * treatment (school group)	1.09	2	8.86	<0.001
Time factor *sex * school group	0.99	2	0.84	0.48
Error	20.34	329	-	-
**Between subject effects**				
Age in months	3.75	1	32.20	<0.001
Height-for-age Z-score	0.99	1	8.50	0.004
Sex	0.14	1	1.20	0.30
Treatment (school group)	0.82	2	3.54	0.030
Sex * school group	0.08	2	0.36	0.70
Error	38.39	329	-	-

## Discussion

An important finding of this study is that VA deficiency at school age is a serious public health problem in the intervention areas, since 47.2% in Kaya and 37.1% in Bogandé had low serum retinol at baseline, whereas the cut-off for a severe public health problem is 20% low serum retinol according to WHO [33]. This confirms previous findings in school-children in Niger [32], with a 45% baseline rate of low serum retinol. In the 15 RPO schools in Kaya and in the 8 RPO schools in Bogandé, the rate of low serum retinol was down to 13% and 15%, respectively, so that the VA deficiency went from a severe to a moderate public health problem, after an average of 28 and 51 RPO fortified meals in Kaya and Bogandé respectively, over a year. These findings are in accordance with previous studies showing the efficacy or effectiveness of RPO among preschool children [27], pupils [34], and reproductive age women [4,25]. As suggested by Wasanwisut [35], the intervention was considered effective since the deficiency rate was down to 15% or less in all intervention groups.

The VA supplied by the RPO supplement over the test year amounted to approximately 42 mg RAE in Kaya and 76.5 mg in Bogandé, which is close to the 60 mg provided by a single VA capsule if we use 6:1 as conversion factor for β-carotene to retinol. Had we used the newly recommended conversion factor of 12:1 [36], the total amount of VA provided as RPO would have represented around half of the dosage of a VA capsule. It is interesting to note that the RPO and the single VA capsule had a nearly equivalent impact on serum retinol in Bogandé school children. RPO was not found more effective than retinol supplements in our study, however, which is at variance with others [34,37]. It may be simply a matter of dosage or duration of the RPO fortification in our study, or else, it may be due to the fact that the interval between VA capsule administration and the endline serum retinol measurement was slightly shorter (5 months) than the interval between the last RPO meal and the endline serum retinol measurement (5.5 – 6 months).

Mean final serum retinol in Kaya pupils was nearly twice as high as that of Bogandé RPO pupils, in spite of the fact that the former had received roughly half as much RPO as the latter. This may reflect the fact that in Kaya, pupils were still receiving RPO supplements when endline blood samples were collected for retinol determination, whereas in Bogandé, RPO supplementation was interrupted for school recess 5.5 to 6 months before blood sampling, so that VA stores could be more depleted.

In Bogandé, the rate of VA deficiency at baseline was lower than in Kaya. This is unquestionably due to the different timing of the survey, which took place in the rainy season with plenty of green leaves and mangoes in Bogandé, and during the dry and lean season in Kaya. This may also be why in Kaya, the few children who had received a VA capsule in the course of the National Micronutrient Days 6 months or more before the baseline study did not have a better VA status compared to other pupils.

In both sites, our findings support previous studies showing that initially deficient subjects derived the most benefit from the VA supplement, whether in the form of RPO or a single VA capsule [4,6,38-40]. In initially deficient pupils of Bogandé, serum retinol increased by 87.5% with the VA capsule and by 63.1% with RPO meals. In VA replete subjects, there was no further increase in serum retinol; there was even a tendency for the reverse, with 12% of the normal pupils at baseline showing a low serum retinol value at endline (VA capsule or RPO treatment). Such paradoxical findings were reported previously, but with synthetic VA, not with food supplements [6,40]. It may simply reflect regression to the mean, but further research on the potential adverse effect of VA supplementation among non-deficient children is warranted.

In Kaya and in Bogandé as well, the rate of low serum retinol remained quite high (between 13% and 17%) after the intervention, however. This high residual rate, whether with the VA capsule or RPO "treatment", again shows that a dosage of approximately 60 mg RAE sustains normal VA status for less than 6 months. Among pre-schoolers of the same Kaya area, Zagré et al [4] had reported that 6 months following a VA capsule distribution among preschoolers with a coverage rate around 90% in Burkina Faso, the rate of low serum retinol was 84.5%. In under-five children of Niger, it was shown that three months following VA capsule administration, the rate of low serum retinol was practically back to baseline level of 38% [11]. Although this was not the purpose of the present study, we could observe that the benefit of VA capsules was indeed short-lived. In one of the intervention sites (Kaya), 18% of the pupils had received a VA capsule 6 months prior to our baseline survey, but their VA status was not different from that of other pupils. So providing some 60 mg of VA either through RPO fortification of school meals or through a single VA capsule over the test year only alleviates the VAD problem in school children. At least two VA capsules per year if not more, or a higher level of RPO fortification of school meals, or a combination of VA supplementation and fortification, would be required.

Factors other than VA intake may also contribute to low serum retinol values, and these should not be overlooked. One of those factors is underlying infection, which is known to reduce serum retinol [41,42] and makes serum retinol non-specific of VA deficiency. We could have used a more sensitive and specific indicator of VA deficiency such as the modified relative dose-response [43-45], but the required retinol analog was only available at high cost. We collected information on the occurrence of illness in the previous fortnight, but this variable showed no significant association with serum retinol at either time. Another factor that may explain the persistence of more than 10% low serum retinol values is the presence of concurrent nutritional deficiencies which may act as limiting factors. Zinc deficiency is widely prevalent among children worldwide, and it is known to affect growth [46] and to interact with VA [47]. The fact that taller children had a higher serum retinol response in both areas indeed suggests that zinc or protein-energy malnutrition may interfere with VA status improvement. These observations lead to advocate for more global nutritional approaches to micronutrient malnutrition rather than single nutrients, and therefore dietary diversification strategies, along with public health measures to control infection.

Boys are reportedly at higher risk of VA deficiency [48-50] although the reasons for their higher vulnerability are largely unexplained. Baseline data did not disclose a better VA status of girls, but their response to RPO in Kaya was significantly higher than that of boys. In Bogandé, sex was not a significant determinant of serum retinol in the co-variance analyses including all three treatment groups. In separate linear regression of endline or change of serum retinol for the capsule and RPO groups, it was found that female sex was associated with a higher response, with the VA capsule but not with RPO supplementation. No explanation for this difference can be proposed.

The interpretations of the findings in Bogandé were obscured by the much better VA status of the negative control group of pupils at baseline. Indeed, the rate of low serum retinol was of the same magnitude as that found in the positive control and RPO groups, but after the intervention. These wide differences underline the disparities that may be found within the population of a relatively small area. Other indices of a better socio-economic status of the negative control group pupils are their significantly higher height-for-age, and the fact that among the 8 schools selected at random to serve as controls, more than half were in villages actively involved in trade. The link between better socio-economic status and better health and nutrition status is well documented [33,51,52]. In all three school groups, BMI was lower at endline than at baseline as the last harvest had been poor, which underlines the vulnerability of the area to food insecurity.

## Conclusion

This study disclosed a high rate of VA deficiency in school children in Burkina Faso. This is an important finding, considering that it is customary to focus on preschoolers and mothers as priority target groups for the improvement of VA status. The study also confirmed the effectiveness of RPO as a food supplement for VA in primary school pupils. RPO is a highly bio-effective source of VA, and its production can be increased even in marginal areas such as Burkina Faso. Further, its distribution could be developed at the regional level, thereby reaching other countries where VA deficiency is a public health problem. RPO is also well liked by West-African populations even if they have not been exposed to it; it was very popular among exposed pupils in this study. Furthermore, palm oil plantations, and the extraction and commercial distribution of RPO, may generate income for women who are the ones producing and selling the oil. The potential benefits of RPO for VA and for other nutritional and economic benefits in Sahelian countries is only beginning to be recognized, and the evaluation of the project in Burkina Faso has allowed to advocate for RPO as part of the national strategy [53].

## Competing interests

The author(s) declare that they have no competing interests.

## Abbreviations

BMI, Body Mass Index; DC, Developing Country; HPLC, High Performance Liquid Chromatography; RAE, Retinol Activity Equivalents; RPO, Red Palm Oil; VA, Vitamin A.

## Authors' contributions

ANZ collected the field data, as part of his research for the M.Sc. (Nutrition). HD designed the study and supervised ANZ. YMP was the co-supervisor of ANZ in the field. ITS was in charge of serum retinol determinations, and he contributed his comments on the paper drafted by ANZ, redrafted (in English) by HD, and reviewed by YMP.
